# Neuromuscular adaptations to load-matched isometric vs. dynamic resistance training in Kho-Kho athletes: a pilot investigation

**DOI:** 10.3389/fspor.2026.1841049

**Published:** 2026-07-14

**Authors:** Zhaoxiang Zhang, Małgorzata Tomicka, Xiaodan Guo, Mingmao Li, Piotr Aschenbrenner

**Affiliations:** 1 Gdansk University of Physical Education and Sport, Gdańsk, Poland; 2 Department of Biomechanics and Sport Engineering, Gdansk University of Physical Education and Sport, Gdansk, Poland

**Keywords:** Kho-Kho, isometric training, dynamic resistance training, perceived exertion, peak torque, sprint acceleration, vertical jump, reactive strength index

## Abstract

**Introduction:**

This pilot study compared the effects of isometric resistance training (ISO) and dynamic resistance training (DYN) on neuromuscular and sport-related performance in collegiate Kho-Kho athletes.

**Methods:**

Seventeen athletes (11 males and 6 females) completed the study and were included in the final analysis: ISO, n = 9 (6 males and 3 females); DYN, n = 8 (5 males and 3 females). Both groups completed a five-week training program, with intensity regulated using modality-specific rating-of-perceived-exertion scales and a target perceived-effort range of 6–7. Pre- and post-intervention assessments included knee extension and flexion torque at multiple angles and velocities, squat linear sprint performance, vertical jump metrics, and reactive strength index.

**Results:**

A significant Group × Time interaction was observed for normalized 75° right knee-extension peak torque, F(1,15) = 6.874, p = .019, *η*²p = .314. Initial squat-start speed improved across groups, F(1,15) = 20.435, p < .001, *η*²p = .577, but the Group × Time interaction was not significant, F(1,15) = 1.083, p = .315, *η*²p = .067. Vertical jump improvement did not show a significant Group × Time interaction, and isokinetic torque outcomes showed no clear modality-specific superiority.

**Discussion:**

These exploratory findings support a possible short-term, joint-angle-specific strength benefit of RPE-regulated isometric training. However, a modality-specific advantage for sprint acceleration was not supported, and definitive superiority over dynamic resistance training cannot be concluded.

## Introduction

Kho-Kho is a high-intensity sport characterized by repeated bouts of sprinting, dodging, diving, and rapid directional changes that demand explosive strength, speed, and agility. Recent Kho-Kho-specific studies have identified speed, agility, physical performance capacity, and role-specific physical attributes as important components of performance in Kho-Kho athletes (1–4). Effective performance requires athletes to generate force rapidly from static or low-position states—such as seated, crouched, or semi-squat positions—to execute quick lunges, acceleration bursts, and evasive movements. This rationale is consistent with Kho-Kho literature emphasizing speed, agility, explosive leg power, and dynamic balance as relevant performance qualities (1, 5). These movement characteristics place substantial demands on knee-extensor and flexor torque production as well as neuromuscular efficiency.

Strength training (ST) modalities are commonly categorized as isometric, isokinetic, and isotonic (6). Torque output across these modalities is influenced by factors such as muscle hypertrophy, mechanical stiffness, muscle length, and biomechanical leverage (7). Prior research indicates that isometric contractions often produce higher peak torque (PT) than isokinetic contractions across a range of joint angles (8). In particular, PT values tend to be optimized at approximately 75° and 45° of knee flexion at isokinetic velocities of 60°/s and 300°/s, respectively (8). These angle-specific findings underscore the need to understand how different contraction modes contribute to functional performance.

Isometric training has been shown to enhance strength measured during both isometric and isokinetic tests. Adaptations differ depending on muscle length, with short-length training typically improving neural drive—reflected in increased electromyographic activity—whereas training at longer lengths tends to promote hypertrophy (9). Isometric contractions can also generate substantially higher absolute torque outputs (33%–75% greater) than dynamic contractions (8), largely because the absence of movement allows maximal tension development during the early contraction phase (50–100 ms) (10). These characteristics suggest that isometric training may offer advantages for improving explosive actions, particularly those initiated from static positions.

Resistance-training prescription can influence strength and hypertrophy adaptations, although responses depend on loading strategy, task demands, and athlete status (11, 12). Strength-training interventions of both isometric and dynamic types have been shown to improve key athletic performance outcomes. Short-term isometric programs (approximately six weeks) can achieve strength and vertical jump improvements comparable to those of dynamic training (8, 13). Meta-analytic evidence further indicates that strength-training interventions lasting more than three weeks yield consistent improvements in sprint speed, agility, and jump performance in adolescent team-sport athletes (14, 15). However, despite these findings, the comparative effects of isometric vs. dynamic training under matched internal load conditions remain unclear—particularly in sports such as Kho-Kho, where explosive force generation from low starting positions is central to performance.

Given these sport-specific demands, identifying effective and practical training methods is essential for coaches and practitioners. No study to date has directly compared isometric and dynamic strength training in Kho-Kho athletes while controlling perceived exertion to standardize training load. Therefore, the present study investigates the comparative effects of isometric vs. dynamic training—each regulated to approximately 75% of maximal voluntary contraction using RPE scales—on key performance outcomes in Kho-Kho players. It was hypothesized that isometric training would elicit greater improvements in peak torque (PT), Squat Linear Sprint (SLS) performance, and vertical jump height compared with dynamic training.

## Materials and methods

### Study design

This study used a 5-week, parallel-group, pre–post pilot design to compare the effects of isometric resistance training and dynamic resistance training on neuromuscular and sport-related performance in Kho-Kho athletes. Because the study was conducted within an active team-training environment, withholding strength training from a subgroup of athletes was considered impractical and potentially disruptive to regular team preparation. Therefore, a passive non-training control group was not included. The absence of a passive control group limits causal inference. Specifically, changes observed after the intervention may partly reflect repeated testing, familiarization with the assessment procedures, normal sport training exposure, or natural short-term adaptation rather than the experimental training modalities alone. For this reason, the present study should be interpreted as an exploratory pilot investigation rather than a definitive efficacy trial. Future studies should include passive or active control groups, or crossover designs, to better isolate the specific effects of isometric and dynamic contraction modalities. All experimental procedures were approved by the Bioethics Committee of the District Medical Chamber, Gdańsk, Poland (Approval KB-22/17) and were conducted in accordance with national legal and ethical standards.

Furthermore, comparative pre–post designs using two intervention groups are widely accepted in applied sports science and have been used extensively in soccer, basketball, and field-sport training studies (16). These designs are appropriate for generating exploratory evidence when ecological validity and athlete availability preclude randomized controlled trials. Therefore, the present study adopted a controlled two-group design with standardized internal training load.

Training load was regulated using modality-specific perceived exertion scales, with the IES used for ISO and the Borg CR-10 scale used for DYN. Both groups were maintained within a target perceived-effort range of 6–7. Thus, the interventions were matched primarily for internal perceived effort rather than identical external mechanical work.

### Participants

Initially, 28 Kho-Kho athletes were recruited and allocated to either the isometric resistance training group (ISO; n = 14; 9 males and 5 females) or the dynamic resistance training group (DYN; *n* = 14; 10 males and 4 females). Seventeen participants completed the full 5-week intervention and all pre- and post-intervention assessments and were included in the final analysis (11 males and 6 females). The final ISO group included 9 participants (6 males and 3 females), and the final DYN group included 8 participants (5 males and 3 females). Eleven participants were not included in the final analysis because of scheduling conflicts or insufficient training attendance. Because attrition was considerable, selection bias cannot be excluded. In addition, because of the small final sample size and limited numbers of males and females within each group, sex was not included as an independent factor in the statistical model. Therefore, sex-specific training responses could not be examined in the present pilot study, and the findings should be interpreted cautiously. Participant characteristics for the final analyzed sample are presented in Table 1.

**Table 1:** Participant characteristics (mean ± SD).

Group	n	Sex (M/F)	Age (y)	Body mass (kg)	Fat-free mass (kg)	Height (cm)
Isometric	9	6/3	20.2 ± 2.2	73.9 ± 16.6	62.0 ± 17.5	176.6 ± 14.1
Dynamic	8	5/3	21.8 ± 1.7	78.3 ± 17.7	64.3 ± 18.2	174.3 ± 13.7

Values are mean ± SD. M, male; F, female.

An *a priori* power analysis was conducted using G*Power 3.1.9.7 for an *F* test repeated-measures ANOVA with a within–between interaction. The analysis was based on an expected medium effect size (f = 0.25), *α* = 0.05, statistical power = 0.80, two groups, two repeated measurements, a correlation among repeated measures of 0.50, and a nonsphericity correction of 1.00. This calculation indicated that a total sample size of 34 participants would be required to detect a medium Group  ×  Time interaction effect with adequate power. Although 28 athletes were initially recruited, the final analyzed sample after attrition was 17 participants (ISO: n = 9; DYN: n = 8), which was below the *a priori* target. Therefore, the present study should be interpreted as an exploratory pilot investigation, and non-significant findings should not be interpreted as clear evidence of no training effect.

All participants reported no existing lower-limb injuries or neuromuscular conditions and refrained from rigorous training activities during the six months preceding the intervention. Body composition was assessed using InBody 720 bioelectrical impedance analysis.

### Rate of perceived exertion (RPE)

The five-week intervention consisted of 10 training sessions. Each session included 12 prescribed exercise exposures, resulting in 120 total exercise exposures across the intervention. Perceived exertion was used to regulate training intensity at a target level corresponding to approximately 75% of maximal voluntary contraction (MVC). Because the contraction modes differed between groups, modality-specific RPE scales were used: the Isometric Exercise Scale (IES) for the ISO group and the Borg CR-10 scale for the DYN group (17–19). Previous evidence indicates a strong correlation between the IES and CR-10 scales (r = 0.97), as well as strong relationships between each scale and measured exercise intensity (IES: r = 0.89; CR-10: r = 0.88; *p* < 0.001). Therefore, these scales were used to maintain a comparable perceived-effort range between groups rather than to equate external mechanical work exactly. Participants completed familiarization sessions before the intervention, and a target RPE of 6–7 was maintained throughout training. For each participant, mean RPE was calculated across all completed exercise exposures. Participant-level mean RPE values were then compared between groups to evaluate whether perceived internal load was comparable across the intervention.

### Borg CR-10 scale format, isometric exercise scale (IES) format

**Figure 1:**
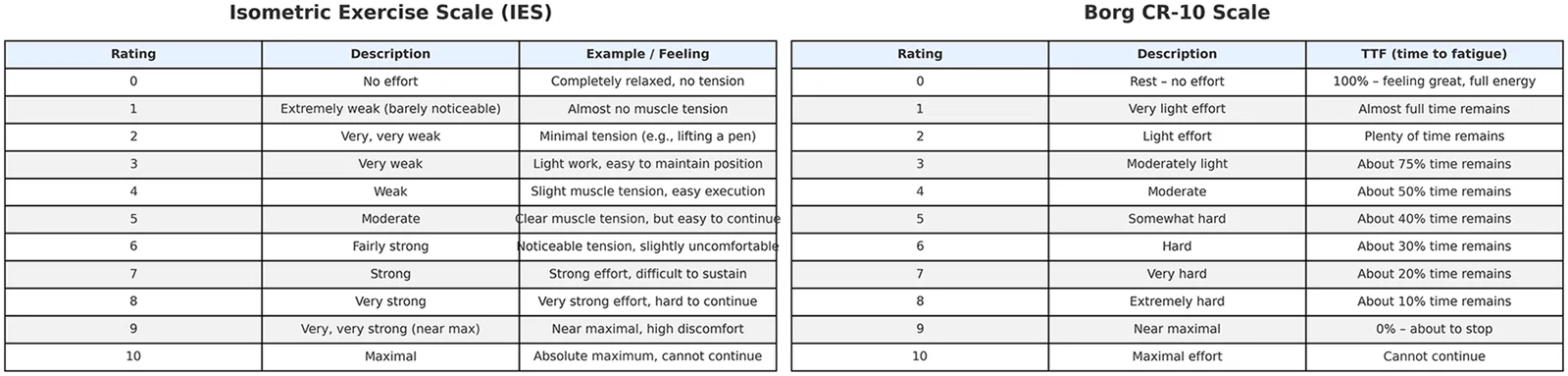
Rating of perceived exertion (RPE) scales used in the study. Left panel: Borg CR-10 scale for the DYN group. Right panel: Isometric Exercise Scale (IES) for the ISO group. Polish translations were provided for comprehension. The red highlight marks the target exertion range (RPE 6–7, ∼75% MVC).

Figure 1: RPE scales used in the study. Left: Borg CR-10 scale for the DYN group. Right: Isometric Exercise Scale (IES) for the ISO group. Polish translations were provided to enhance participant comprehension. The red highlight marks the target exertion level (6–7, corresponding to ∼75% MVC).

### Peak torque test

PT was quantified bilaterally for the knee extensors and flexors using a Biodex System 4 Pro dynamometer (version 4.2 software). Before testing, participants completed a 10-minute standardized warm-up consisting of low-intensity lower-limb movements and dynamic mobility exercises, followed by familiarization trials for each testing condition. Participants were seated on the dynamometer chair with the hip and thigh stabilized using straps, and the dynamometer axis was aligned with the lateral femoral epicondyle of the tested limb. Gravity correction was performed according to the manufacturer's procedures before each testing condition. The same limb testing order was maintained for each participant at pre- and post-testing to minimize order effects.

Isometric assessments were performed at fixed knee angles of 75° and 45°. Each isometric contraction lasted 5 s, with 10 s between trials and 20 s between angles. Isokinetic assessments were performed at angular velocities of 60°/s and 300°/s, with each test consisting of five maximal repetitions. Participants received standardized verbal encouragement during all maximal efforts. For each testing condition, the highest valid peak torque value was retained for analysis. The Biodex System 4 has shown good-to-excellent reliability for lower-limb isokinetic strength testing in physically active adults and good reliability for knee flexion and extension at 60°/s in youth populations (20, 21). Sample-specific reliability indices were not calculated in the present cohort.

### Normalized peak torque

Individual variation in response to strength training has been previously reported, with some individuals showing greater metabolic or enzymatic adaptations than others despite similar group-level mechanical or hypertrophic responses (22). Because the present sample included young, physically active athletes, who may have relatively limited scope for learning- or coordination-related improvement compared with untrained individuals, we followed previous strength-training work involving healthy active participants (8). To reduce the influence of body size and body composition on peak torque (PT), PT values were normalized to fat-free mass (FFM) using allometric scaling.

Allometric scaling assumes that strength does not increase in a simple linear proportion to body size (13, 23). Therefore, normalized strength can be expressed as:

$$\mathrm{Normalized}\;\;\mathrm{strength}=\mathrm{Strength}/\mathrm{Body}\;\;\mathrm{siz}e^{b}$$

For torque outcomes, a higher scaling exponent may be required than for force because torque is influenced not only by muscle force production but also by limb length, lever arms, and body-size-related mechanical factors (13, 24, 25). In the present study, normalized peak torque was calculated using an exponent of 1.12:

$$\mathrm{Normalized}\;\;\mathrm{peak}\;\;\mathrm{torque}=\mathrm{Peak}\;\;\mathrm{torque}/\mathrm{FF}M^{1.12}$$

where peak torque was expressed in N·m and FFM in kg. This approach was used to improve comparability between participants with different body-composition profiles and to reduce potential confounding from differences in fat-free mass.

### Optojump test

Performance assessments were conducted using the OptoJump Next system [v1.7.0 (26);] combined with a GYKO Pro inertial device (27) to evaluate sport-related lower-limb performance variables, including sprint speed, jump height, power output, contact time, velocity, and reactive strength index (RSI). The OptoJump system has demonstrated strong concurrent validity and excellent test–retest reliability for vertical jump height assessment, with previously reported validity ICCs of 0.997–0.998 and test–retest ICCs of 0.982–0.989 (28). OptoJump Next has also been reported to provide reliable temporal measures such as contact and flight time, although system settings may influence measurement precision and should be considered when interpreting temporal variables (29).

Participants performed two sport-related performance tests. The Squat Linear Sprint (SLS) consisted of an 8-m sprint initiated from a squat position and was selected because Kho-Kho frequently requires rapid acceleration from low, crouched, or semi-static positions. The BFS vertical jump was performed as a maximal-effort vertical jump and was used to assess lower-limb explosive performance, including jump height, power output, contact time, velocity, and RSI. Although these tests reflect important physical components of Kho-Kho performance, they should be considered sport-related rather than fully validated Kho-Kho-specific tests, as they do not fully capture multidirectional chasing, dodging, reactive decision-making, or rapid change-of-direction demands during match play.

### Training protocol

Training sessions were conducted twice per week for five weeks, for a total of 10 sessions. Each session included three exercises, with each exercise performed for four sets. Rest intervals were standardized at 1 min between sets and 2 min between exercises. Each session began with a standardized warm-up consisting of dynamic mobility exercises, glute activation, bodyweight squats or lunges, and low-intensity plyometric movements. After the training exercises, participants completed a cool-down including low-intensity movement, static stretching, and recovery-focused mobility exercises. Training intensity was regulated using the target RPE range of 6–7, corresponding to approximately 75% MVC, and perceived exertion was recorded immediately after each exercise. Details of the training protocol are presented in Table 2.

**Table 2:** Training program (5 weeks; 2 sessions/week; ∼40 min/session).

Component	Isometric group	Dynamic group
Warm-up (10 min)	Dynamic mobility, glute activation, plyometric hops	Mobility drills, bodyweight squats, lunges
Exercise 1	Isometric knee extension curl (75%–90% MVC; 6–8 s; 4 sets)	Dynamic leg press (load adjusted to CR-10 RPE 6–7; 6–8 reps; 4 sets)
Exercise 2	Isometric squat (hold 6–8 s; 4 sets)	Fixed Barbell squat (load adjusted to CR-10 RPE 6–7; 6–8 reps; 4 sets)
Exercise 3	Wall sit (maximal effort; 6–8 s; 4 sets)	Bulgarian split squat (6–8 reps; 4 sets)
Rest	1 min between sets; 2 min between exercises	1 min between sets; 2 min between exercises
Cool-down (10 min)	Foam rolling, static stretching	Proprioceptive balance drills, static stretching

ISO, isometric resistance training group; DYN, dynamic resistance training group. The term “isokinetic” is used only for Biodex testing outcomes measured at fixed angular velocities and does not describe the DYN training intervention.

### Statistical analysis

Statistical analyses were conducted using IBM SPSS Statistics 27. Data are reported as mean ± standard deviation for normally distributed variables and median ± interquartile range (IQR) for non-normally distributed variables. Normality was assessed using the Shapiro–Wilk test, supported by inspection of skewness and kurtosis.

For normally distributed outcomes, two-way repeated-measures ANOVA was used with Time as the within-subject factor and Group as the between-subject factor. Main effects of Time and Group and the Group  × Time interaction were reported as F-values, degrees of freedom, p-values, and partial eta squared (*η*^2^*p*). The Group × Time interaction was used as the primary indicator of modality-specific adaptation. Partial eta squared was interpreted as small = 0.01, medium = 0.06, and large ≥0.14.

Within-group changes were analyzed using paired-samples t-tests for normally distributed variables and Wilcoxon signed-rank tests for non-normally distributed variables. Cohen's d was reported for parametric comparisons (30) and interpreted as small = 0.20, medium = 0.50, and large ≥0.80. For Wilcoxon tests, effect size r was calculated as |Z|/√N and interpreted as small = 0.10, medium = 0.30, and large ≥ 0.50. Statistical significance was set at *p* < 0.05. Because multiple outcomes were analyzed, p-values were not formally adjusted and were interpreted together with effect sizes, confidence intervals, and Group × Time interaction results.

A *post-hoc* sensitivity estimate was calculated using the same G*Power model as the *a priori* analysis. With the final sample of 17 participants, achieved power to detect a medium Group  ×  Time interaction effect was approximately 0.49. Therefore, results were interpreted primarily using effect sizes, confidence intervals, and the pilot-study context rather than p-values alone.

## Results

### RPE values

Recorded RPE values confirmed comparable perceived effort between groups. Participant-level mean RPE was 6.60 ± 0.45 in the ISO group and 6.61 ± 0.58 in the DYN group, with no significant between-group difference (Mann–Whitney U = 33.0, *p* = .815).

### Isometric peak torque

**Figure 2:**
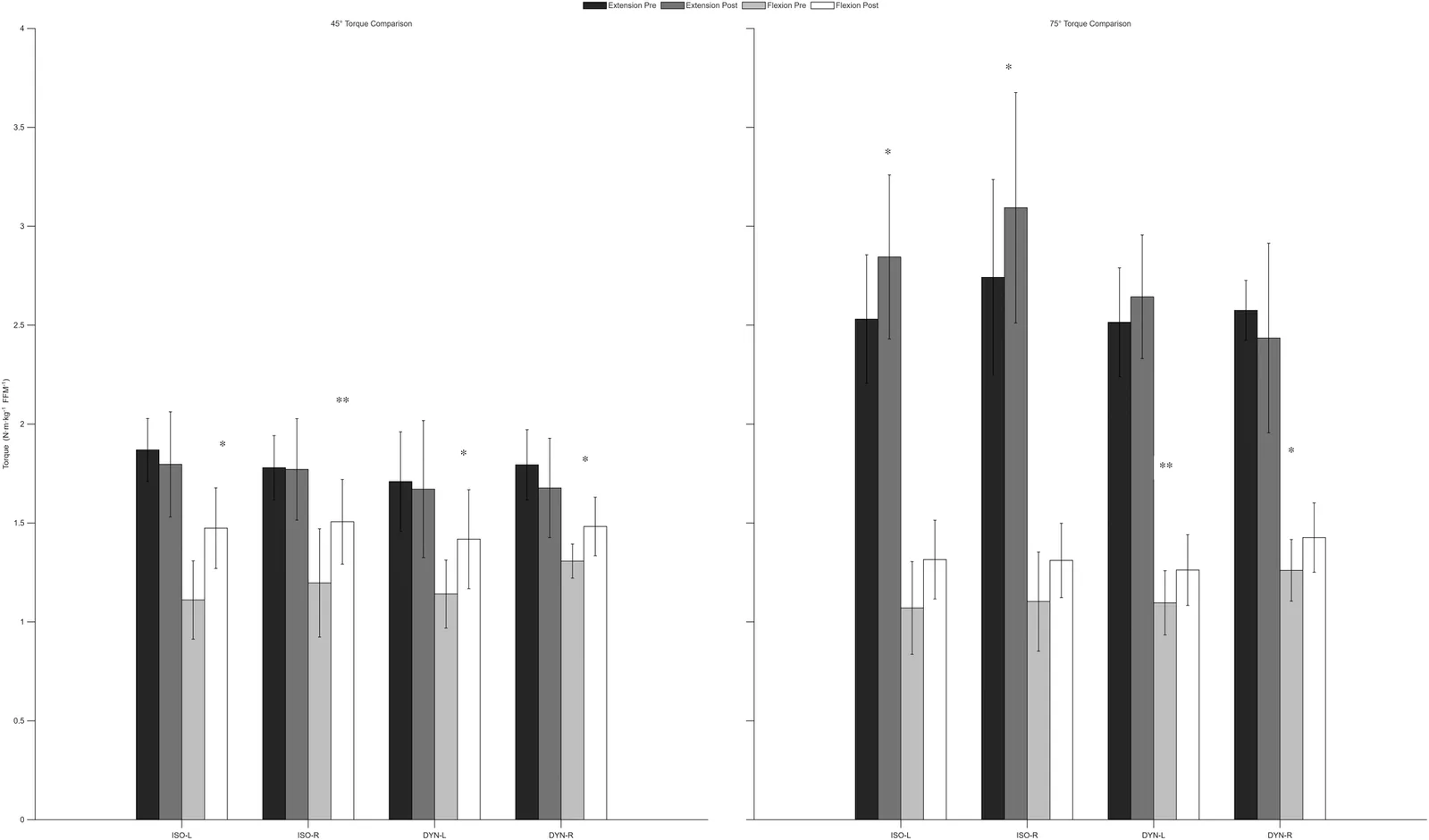
Isometric normalized peak torque (NPT) at 45° and 75° knee joint angles. Bars show mea*n* ± SD values pre- and post-training for extension and flexion, left and right limbs. Asterisks indicate significant within-group improvements (*p* < 0.05). Torque values are normalized to fat-free mass (N·m·kg^−1.12^_FFM_).

For isometric testing, normalized peak torque (NPT) results are summarized in Table 3 and illustrated in Figure 2. At 75° knee extension, the ISO group improved on the left (−0.335 ± 0.189, p = .001, d_z = −1.769) and right (−0.283 ± 0.315, p = .027, d_z = −0.900), whereas the DYN group did not show significant within-group changes. For PT 75° L EX, the main effect of Time was significant, F(1,15) = 17.132, p = .001, η²p = .533, whereas the Group × Time interaction was not significant, F(1,15) = 3.378, p = .086, η²p = .184. For PT 75° R EX, the Group × Time interaction was significant, F(1,15) = 6.874, p = .019, η²p = .314, indicating a larger change in ISO than DYN. At 45° knee extension, no significant Time, Group, or Group × Time effects were observed.

**Table 3:** Normalized isometric peak torque and repeated-measures ANOVA.

Outcome	Group	Change/Z	SD	Within-group p	Effect size	95% CI	Time effect	Group effect	Group×Time interaction
PT 75° L EX	ISO	−0.335	0.189	.001	dz = −1.769	[−2.823, −0.678]	F(1,15) = 17.132 p = .001 *η*2p = .533	F(1,15) = 0.345 p = .566 *η*2p = .022	F(1,15) = 3.378 p = .086 *η*2p = .184
	DYN	−0.129	0.270	.219	d_z_ = −0.477	[−1.197, 0.273]			
PT 75° R EX	ISO	−0.283	0.315	.027	d_z_ = −0.900	[−1.665, −0.097]	F(1,15) = 0.794 p = .387 η^2^p = .050	F(1,15) = 3.280 p = .090 η^2^p = .179	F(1,15) = 6.874 p = .019 η^2^p = .314
	DYN	0.140	0.350	.297	d_z_ = 0.398	[−0.337, 1.108]			
PT 75° L Flex	ISO	−0.252	0.209	.007	d_z_ = −1.207	[−2.061, −0.313]	F(1,15) = 29.528 p < .001 η^2^p = .663	F(1,15) = 0.222 p = .644 η^2^p = .015	F(1,15) = 1.258 p = .280 η^2^p = .077
	DYN	−0.166	0.062	<.001	d_z_ = −2.677	[−4.197, −1.127]			
PT 75° R Flex	ISO	−0.203	0.137	.002	d_z_ = −1.480	[−2.426, −0.494]	F(1,15) = 27.936 p < .001 η^2^p = .651	F(1,15) = 1.086 p = .314 η^2^p = .068	F(1,15) = 0.289 p = .599 η^2^p = .019
	DYN	−0.166	0.151	.017	d_z_ = −1.101	[−1.972, −0.184]			
PT 45° L EX	ISO	0.126	0.286	.221	d_z_ = 0.442	[−0.257, 1.117]	F(1,15) = 1.108 p = .309 η^2^p = .069	F(1,15) = 2.471 p = .137 η^2^p = .141	F(1,15) = 0.317 p = .582 η^2^p = .021
	DYN	Z = −1.400	—	.161	r = .495	—			
PT 45° R EX	ISO	0.041	0.205	.567	d_z_ = 0.199	[−0.467, 0.853]	F(1,15) = 1.906 p = .188 η^2^p = .113	F(1,15) = 0.335 p = .571 η^2^p = .022	F(1,15) = 0.444 p = .515 η^2^p = .029
	DYN	Z = −1.400	—	.161	r = .495	—			
PT 45° L Flex	ISO	−0.343	0.224	.002	d_z_ = −1.536	[−2.503, −0.530]	F(1,15) = 25.002 p < .001 η^2^p = .625	F(1,15) = 0.100 p = .757 η^2^p = .007	F(1,15) = 1.491 p = .241 η^2^p = .090
	DYN	−0.209	0.231	.038	d_z_ = −0.902	[−1.714, −0.047]			
PT 45° R Flex	ISO	−0.291	0.114	<.001	d_z_ = −2.563	[−3.942, −1.155]	F(1,15) = 60.379 p<.001 η^2^p = .801	F(1,15) = 0.082 p = .779 η^2^p = .005	F(1,15) = 3.718 p = .073 η^2^p = .199
	DYN	−0.175	0.134	.008	d_z_ = −1.308	[−2.251, −0.321]			

ISO = isometric resistance training group; DYN = dynamic resistance training group; PT = peak torque; EX = extension; Flex = flexion; ES = effect size; CI = confidence interval; η^2^p = partial eta squared. Change values were calculated as pre-intervention minus post-intervention values; therefore, negative values indicate an increase from pre to post for torque outcomes. For normally distributed outcomes, within-group changes are reported as mean change ± SD with Cohen's d_z and 95% CI. For non-normally distributed outcomes, Wilcoxon Z and effect size r are reported without an unbounded CI.

For knee flexion, significant Time effects were observed for PT 75° L Flex, F(1,15) = 29.528, *p* < .001, *η*^2^*p* = .663; PT 75° R Flex, F(1,15) = 27.936, *p* < .001, *η*^2^*p* = .651; PT 45° L Flex, F(1,15) = 25.002, *p* < .001, *η*^2^*p* = .625; and PT 45° R Flex, F(1,15) = 60.379, *p* < .001, *η*^2^*p* = .801. These findings indicate overall pre–post improvements in flexion torque across participants. However, the Time × Group interactions were not statistically significant for flexion outcomes, although PT 45° R Flex approached significance, F(1,15) = 3.718, *p* = .073, *η*^2^*p* = .199. Therefore, flexion improvements should be interpreted primarily as general training-related changes rather than clear evidence of modality-specific superiority.

Overall, the most robust between-group finding for isometric torque was the significant Time × Group interaction for PT 75° R EX. Other significant within-group or Time effects indicate meaningful pre–post changes, but they should be interpreted cautiously unless supported by a significant interaction effect.

### Isokinetic peak torque

**Figure 3:**
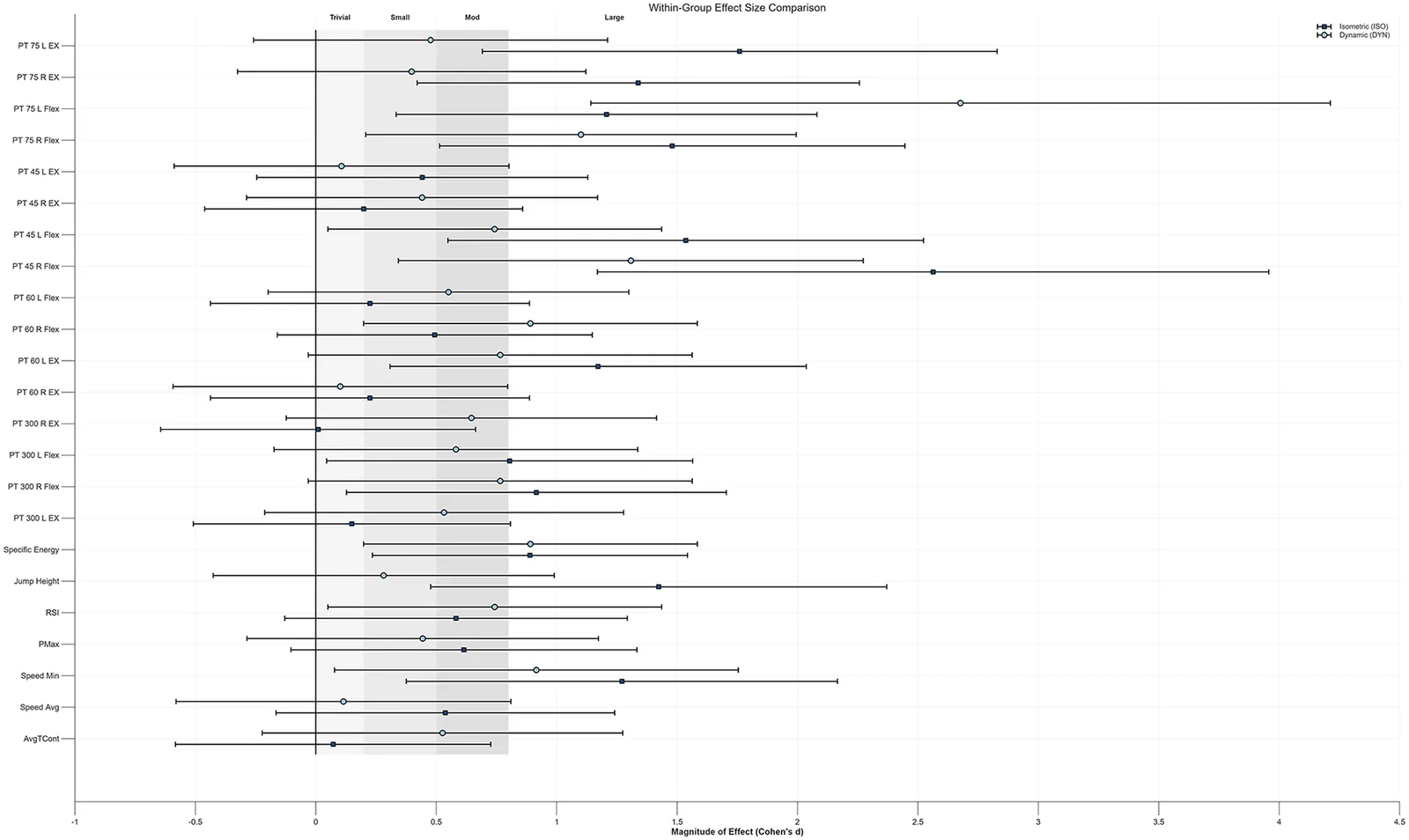
Isokinetic normalized peak torque (NPT) at angular velocities of 60°·s⁻^1^ and 300°·s⁻^1^. Bars represent SD values pre- and post-training for extension and flexion, left and right limbs. Asterisks denote significant within-group changes (*p* < 0.05).

Isokinetic NPT results at 60°/s and 300°/s are summarized in Table 4 and illustrated in Figure 3. Full repeated-measures ANOVA showed a significant main effect of Time for PT 60°/s L EX, F(1,15) = 14.730, *p* = .002, *η*^2^*p* = .495, indicating an overall pre–post change across both groups. However, the Time  ×  Group interaction was not significant, F(1,15) = 0.028, *p* = .870, *η*^2^*p* = .002, suggesting that the magnitude of change did not differ between ISO and DYN.

**Table 4:** Normalized isokinetic peak torque and repeated-measures ANOVA.

Outcome	Group	Change/Z	SD	Within-group p	Effect size	95% CI	Time effect	Group effect	Group×Time interaction
PT 60°/s L EX	ISO	0.194	0.165	.008	dz = 1.172	[0.288, 2.015]	F(1,15) = 14.730 p = .002 η2p = .495	F(1,15) = 0.108 p = .747 η2p = .007	F(1,15) = 0.028 p = .870 η2p = .002
	DYN	0.177	0.232	.067	d_z_ = 0.766	[−0.051, 1.543]			
PT 60°/s R EX	ISO	−0.044	0.195	.519	d_z_ = −0.225	[−0.880, 0.444]	F(1,15) = 0.010 p = .920 η^2^p = .001	F(1,15) = 0.024 p = .879 η^2^p = .002	F(1,15) = 0.373 p = .551 η^2^p = .024
	DYN	0.031	0.308	.781	d_z_ = 0.102	[−0.596, 0.794]			
PT 60°/s L Flex	ISO	0.033	0.146	.519	d_z_ = 0.225	[−0.444, 0.880]	F(1,15) = 0.485 p = .497 η^2^p = .031	F(1,15) = 0.205 p = .657 η^2^p = .013	F(1,15) = 2.586 p = .129 η^2^p = .147
	DYN	−0.083	0.150	.163	d_z_ = −0.551	[−1.283, 0.214]			
PT 60°/s R Flex	ISO	−0.047	0.091	.162	d_z_ = −0.514	[−1.199, 0.199]	F(1,15) = 3.842 p = .069 η^2^p = .204	F(1,15) = 0.020 p = .888 η^2^p = .001	F(1,15) = 0.104 p = .752 η^2^p = .007
	DYN	Z = −1.120	—	.263	r = .396	—			
PT 300°/s L EX	ISO	−0.015	0.102	.665	d_z_ = −0.150	[−0.803, 0.512]	F(1,15) = 1.934 p = .185 η^2^p = .114	F(1,15) = 0.824 p = .378 η^2^p = .052	F(1,15) = 0.584 p = .457 η^2^p = .037
	DYN	−0.053	0.099	.175	d_z_ = −0.533	[−1.262, 0.228]			
PT 300°/s R EX	ISO	0.001	0.089	.977	d_z_ = 0.010	[−0.644, 0.663]	F(1,15) = 1.982 p = .180 η^2^p = .117	F(1,15) = 0.169 p = .686 η^2^p = .011	F(1,15) = 2.089 p = .169 η^2^p = .122
	DYN	−0.066	0.102	.110	d_z_ = −0.646	[−1.397, 0.140]			
PT 300°/s L Flex	ISO	−0.063	0.078	.042	d_z_ = −0.805	[−1.547, −0.027]	F(1,15) = 8.219 p = .012 η^2^p = .354	F(1,15) = 1.630 p = .221 η^2^p = .098	F(1,15) = 0.594 p = .453 η^2^p = .038
	DYN	−0.036	0.062	.144	d_z_ = −0.582	[−1.320, 0.190]			
PT 300°/s R Flex	ISO	−0.096	0.105	.025	d_z_ = −0.916	[−1.686, −0.109]	F(1,15) = 11.904 p = .004 η^2^p = .442	F(1,15) = 1.098 p = .311 η^2^p = .068	F(1,15) = 0.460 p = .508 η^2^p = .030
	DYN	−0.065	0.084	.067	d_z_ = −0.766	[−1.542, 0.051]			

ISO = isometric resistance training group; DYN = dynamic resistance training group; PT = peak torque; EX = extension; Flex = flexion; ES = effect size; CI = confidence interval; η^2^p = partial eta squared. Change values were calculated as pre-intervention minus post-intervention values. Cohen's d_z and 95% CI are reported for parametric comparisons; Wilcoxon Z and r are reported for nonparametric comparisons.

At 300°/s, significant main effects of Time were observed for left flexion, F(1,15) = 8.219, *p* = .012, *η*^2^*p* = .354, and right flexion, F(1,15) = 11.904, *p* = .004, *η*^2^*p* = .442. Within-group analyses showed significant improvements in the ISO group for 300°/s flexion bilaterally (left: −0.063 ± 0.078, *p* < .05; right: −0.096 ± 0.105, *p* < .05). However, the Time × Group interactions were not significant for left flexion, F(1,15) = 0.594, *p* = .453, *η*^2^*p* = .038, or right flexion, F(1,15) = 0.460, *p* = .508, *η*^2^*p* = .030. Therefore, although selected isokinetic outcomes changed over time, these findings should be interpreted as general training-related responses rather than clear evidence of modality-specific superiority of ISO over DYN.

### Peak torque between groups

Between-group effects for isometric NPT outcomes are presented in Table 3. The only statistically significant Time × Group interaction was observed for PT 75° R EX, F(1,15) = 6.874, p = .019, η²p = .314, indicating that the magnitude of change differed between ISO and DYN for this outcome. PT 75° L EX showed a non-significant trend toward a Time × Group interaction, F(1,15) = 3.378, p = .086, η²p = .184. Similarly, PT 45° R Flex showed a large but non-significant interaction effect, F(1,15) = 3.718, p = .073, η²p = .199. Therefore, although several outcomes showed significant within-group or Time effects, clear modality-specific superiority was supported only for PT 75° R EX.

### Vertical jump

Vertical jump outcomes are presented in Table 5. Jump height showed a significant main effect of Time, F(1,15) = 5.231, *p* = .037, *η*^2^*p* = .259, indicating an overall pre–post improvement across both groups. Within-group analysis showed that jump height improved significantly in the ISO group (−4.23 ± 2.97 cm, *p* < .05, d = −1.42), whereas the DYN group showed a smaller, non-significant change (−2.23 ± 7.89 cm, *p* = .451, d = −0.28). However, the Time  ×  Group interaction was not significant, F(1,15) = 0.506, *p* = .488, *η*^2^*p* = .033, indicating that the magnitude of jump-height change did not differ significantly between ISO and DYN.

**Table 5:** Vertical jump outcomes and repeated-measures ANOVA.

Outcome	Group	Change/Z	SD	Within-group p	Effect size	95% CI	Time effect	Group effect	Time × Group interaction
Jump height (cm)	ISO	−4.233	2.974	<.05	d = −1.424	[−2.350, −0.457]	F(1,15) = 5.231; *p* = .037; *η*^2^*p* = .259	F(1,15) = 0.006; *p* = .938; *η*^2^*p* = .000	F(1,15) = 0.506; *p* = .488; *η*^2^*p* = .033
	DYN	−2.225	7.891	.451	d = −0.282	[−0.980, 0.435]			
RSI (m/s)	ISO	−0.043	0.075	.119	d = −0.582	[−1.278, 0.144]	F(1,15) = 7.668; *p* = .014; *η*^2^*p* = .338	F(1,15) = 0.024; *p* = .880; *η*^2^*p* = .002	F(1,15) = 0.645; *p* = .434; *η*^2^*p* = .041
	DYN	Z = 2.100	0.070	<.05	r = .743	[0.050, 1.436]			
Fmax (N/kg)	ISO	Z = 0.178	30.430	.859	r = .059	[−0.594, 0.713]	F(1,15) = 0.003; *p* = .960; *η*^2^*p* = .000	F(1,15) = 1.424; *p* = .251; *η*^2^*p* = .087	F(1,15) = 0.052; *p* = .823; *η*^2^*p* = .003
	DYN	−0.748	12.269	.868	d = −0.061	[−0.752, 0.635]			
Pmax (W/kg)	ISO	−13.887	22.569	.102	d = −0.615	[−1.317, 0.118]	F(1,15) = 4.727; *p* = .046; *η*^2^*p* = .240	F(1,15) = 1.140; *p* = .303; *η*^2^*p* = .071	F(1,15) = 0.647; *p* = .434; *η*^2^*p* = .041
	DYN	−6.386	14.386	.250	d = −0.444	[−1.159, 0.300]			

ISO, isometric resistance training group; DYN, dynamic resistance training group; RSI, reactive strength index; Fmax, maximum force; Pmax, peak power; CI, confidence interval; *η*^2^*p*, partial eta squared. Change values were calculated as pre-intervention minus post-intervention. Negative values therefore indicate an increase from pre to post for outcomes where higher values represent better performance. For normally distributed outcomes, within-group changes are reported as mean change ± SD with Cohen's d. For non-normally distributed outcomes, Wilcoxon Z values are reported with effect size r. Repeated-measures ANOVA effects are reported as F(1,15), p, and *η*^2^*p*. The Time × Group interaction was used as the primary test of modality-specific adaptation.

A significant main effect of Time was also observed for RSI, F(1,15) = 7.668, *p* = .014, *η*^2^*p* = .338, and Pmax, F(1,15) = 4.727, *p* = .046, *η*^2^*p* = .240, whereas Fmax did not show a significant Time effect, F(1,15) = 0.003, *p* = .960, *η*^2^*p* < .001. No significant Group effects or Time  ×  Group interactions were observed for any vertical jump outcome. Therefore, although selected jump-related variables improved over time, the results do not provide clear evidence of modality-specific superiority of ISO over DYN for vertical jump performance.

### Squat linear sprint

Sprint performance results are presented in Table 6. Both groups demonstrated significant within-group improvements in initial speed from the squat-start position (ISO: −0.721 ± 0.567 m/s, p = .005, d_z = −1.271; DYN: −0.451 ± 0.493 m/s, p = .036, d_z = −0.916). The main effect of Time was significant, F(1,15) = 20.435, p < .001, η²p = .577, indicating an overall pre-post improvement. However, neither the main effect of Group, F(1,15) = 0.961, p = .342, η²p = .060, nor the Group × Time interaction, F(1,15) = 1.083, p = .315, η²p = .067, was significant. Therefore, the data support improvement in both groups but do not support a modality-specific advantage for initial squat-start speed.

**Table 6:** Squat linear sprint outcomes and repeated-measures ANOVA summary.

Outcome	Group	Change/Z	SD	Within-group p	Effect size	95% CI	Time effect	Group effect	Group×Time interaction
Initial speed (m/s)	ISO	−0.721	0.567	.005	dz = −1.271	[−2.146, −0.356]	F(1,15) = 20.435; p < .001; η2p = .577	F(1,15) = 0.961; p = .342; η2p = .060	F(1,15) = 1.083; p = .315; η2p = .067
	DYN	−0.451	0.493	.036	d_z_ = −0.916	[−1.732, −0.057]			
Average speed (m/s)	ISO	−0.177	0.329	.145	d_z_ = −0.538	[−1.226, 0.179]	F(1,15) = 1.170; p = .296; η^2^p = .072	F(1,15) = 0.051; p = .824; η^2^p = .003	F(1,15) = 2.129; p = .165; η^2^p = .124
	DYN	0.026	0.228	.754	d_z_ = 0.115	[−0.584, 0.806]			
Specific energy (J/kg)	ISO	Z = −0.652	—	.515	r = .217	—	F(1,15) = 0.001; p = .971; η^2^p < .001	F(1,15) = 0.768; p = .395; η^2^p = .049	F(1,15) = 0.493; p = .493; η^2^p = .032
	DYN	0.034	0.129	.475	d_z_ = 0.267	[−0.449, 0.964]			
Average contact time (s)	ISO	Z = −0.830	—	.407	r = .277	—	F(1,15) = 0.698; p = .417; η^2^p = .044	F(1,15) = 0.076; p = .787; η^2^p = .005	F(1,15) = 1.321; p = .268; η^2^p = .081
	DYN	Z = −1.400	—	.161	r = .495	—			

ISO = isometric resistance training group; DYN = dynamic resistance training group; ES = effect size; CI = confidence interval; η^2^p = partial eta squared. Change values were calculated as pre-intervention minus post-intervention values. Negative initial-speed changes therefore indicate higher post-intervention speed.

No significant Time, Group, or Group × Time effects were observed for average sprint speed, specific energy, or average contact time. For average speed, the Time effect was F(1,15) = 1.170, p = .296, η²p = .072, and the Group × Time interaction was F(1,15) = 2.129, p = .165, η²p = .124. For specific energy, the Group × Time interaction was F(1,15) = 0.493, p = .493, η²p = .032; for average contact time, it was F(1,15) = 1.321, p = .268, η²p = .081. Thus, the sprint findings indicate a general improvement in initial speed across groups, whereas average speed, specific energy, and contact time were not significantly altered.

### Standardized mean difference (SMD)

**Figure 4:**
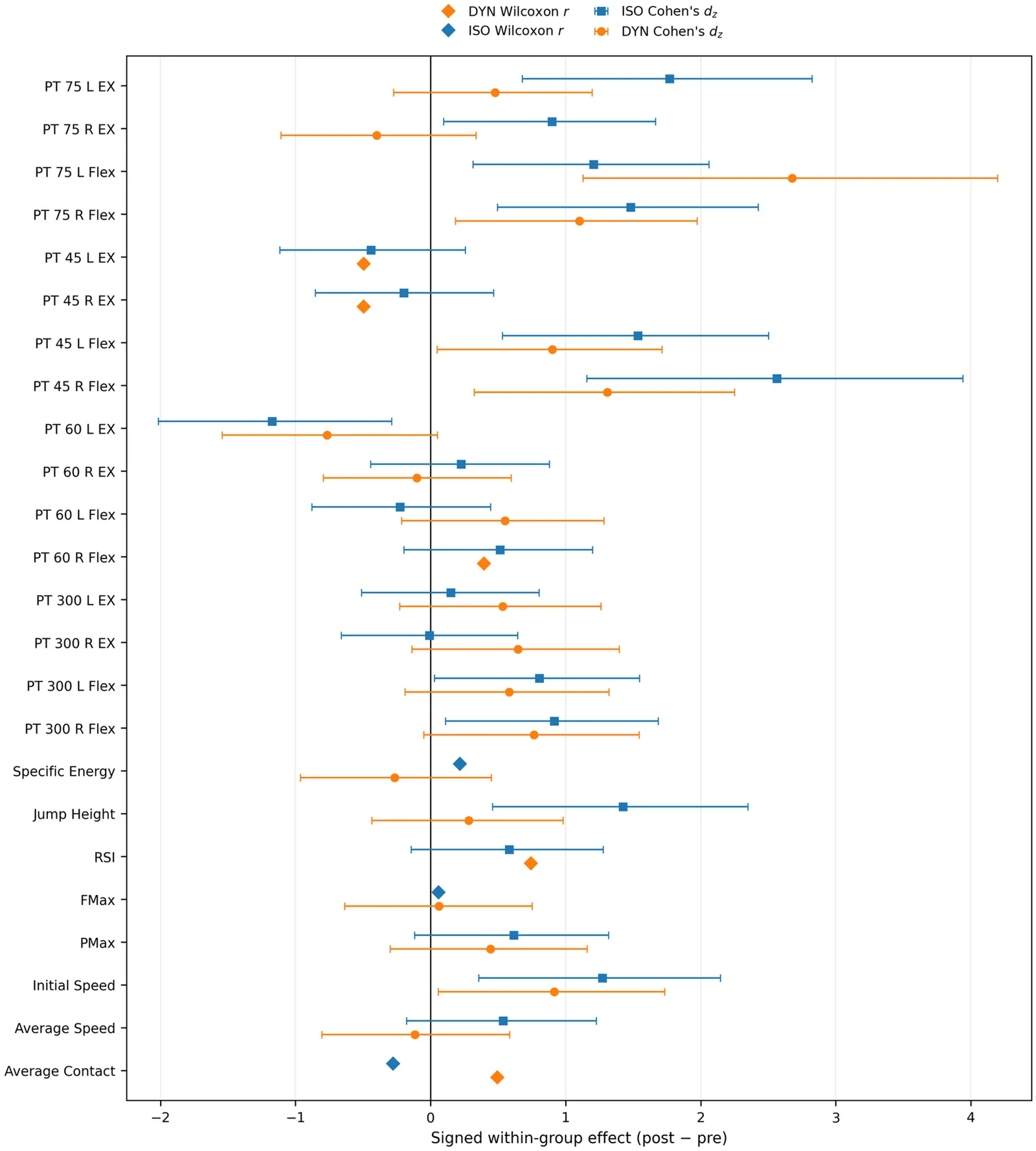
Signed within-group effects for the Isometric (ISO) and Dynamic (DYN) groups. For outcomes analyzed parametrically, Cohen's d_z was calculated as (post-intervention − pre-intervention)/SD of the paired change and is shown with 95% confidence intervals. Outcomes analyzed with Wilcoxon signed-rank tests are shown as signed effect size r without confidence intervals. The sign indicates the direction of the post-minus-pre change; the magnitude of r is not directly comparable with d_z. The jump-height, RSI, FMax, and PMax effect estimates were retained from the published summary statistics because participant-level jump data were unavailable for re-estimation. The plot is descriptive; modality-specific differences should be judged from the Group × Time interaction tests.

Figure 4 presents signed within-group effects for the ISO and DYN groups. For parametrically analyzed outcomes, points represent Cohen's d_z calculated as (post-intervention − pre-intervention)/SD of the paired change, with 95% confidence intervals. Outcomes analyzed with Wilcoxon signed-rank tests are shown as signed r values without confidence intervals. Positive values indicate an increase from pre to post, whereas negative values indicate a decrease; the practical meaning of the direction depends on the outcome. The jump-height, RSI, FMax, and PMax points are retained from the published summary statistics because the participant-level jump dataset was not available for re-estimation. Because r and d_z are different effect-size metrics, their magnitudes should not be compared directly.

The plot is descriptive and must be interpreted together with the Group × Time interaction tests. The corrected analyses support a differential response for PT 75° R EX. Initial squat-start speed improved in both groups without a significant Group × Time interaction, and vertical jump improvement likewise did not show a significant Group × Time interaction. Therefore, Figure 4 does not establish modality-specific superiority for sprint or jump performance.

## Discussion

This study aimed to evaluate the effects of isometric and dynamic strength training (ST) on sport-related performance measures in Kho-Kho athletes. Given the scarcity of research on evidence-based training strategies for Kho-Kho athletes, the present findings provide preliminary but meaningful insights into how contraction type and controlled load via RPE may influence key athletic attributes, including torque production, sprint performance, and jump height. Because both interventions were regulated using modality-specific RPE scales, the design reduced differences in perceived effort between groups and allowed a more balanced comparison of contraction mode.

Both training modalities were standardized to approximately 75% MVC, a level previously shown to provide sufficient neuromuscular stimulus without inducing excessive fatigue (31, 32). This approach may have supported comparable perceived effort across the intervention while reducing fatigue-related confounding. Although this approach supports internal validity, the relatively narrow RPE range may also partially explain why several outcomes improved similarly in both groups.

### Isometric torque improvements

The ISO group showed a greater improvement in normalized peak torque (NPT) at 75° knee extension, particularly on the right side, where the Time  ×  Group interaction was significant. This finding is consistent with the joint-angle specificity of isometric training, where adaptations are often greatest near the trained angle (33, 34). Previous studies have suggested that isometric training at longer muscle lengths, such as approximately 75°, may promote favorable strength adaptations and, over longer periods, may contribute to hypertrophic or architectural changes compared with shorter-length training (9, 35, 36). However, because the present intervention lasted only five weeks and did not include muscle imaging or EMG, the observed improvements are more cautiously interpreted as joint-angle-specific strength adaptations rather than confirmed hypertrophic or neural changes.

Both groups showed meaningful flexion-torque improvements at 75° and 45°, with several significant Time effects. These findings suggest that both training approaches provided a sufficient stimulus for hamstring strength development. The sustained high-tension contractions used during isometric training may have contributed to improved force production capacity, but mechanisms such as motor-unit synchronization, posterior-chain activation efficiency, or neural drive were not directly measured. Therefore, these explanations should be regarded as plausible mechanisms rather than confirmed findings, especially because most flexion outcomes did not show significant Time  ×  Group interactions.

### Isokinetic torque and velocity-specific adaptations

Isokinetic torque adaptations were less clearly modality-specific than the isometric torque outcomes. At 60°·s⁻^1^, selected outcomes showed pre–post changes, but no significant Time  ×  Group interaction was observed, indicating that changes did not differ clearly between ISO and DYN. At the faster velocity of 300°·s⁻^1^, the ISO group showed significant within-group improvements in flexion torque bilaterally; however, the Time  ×  Group interactions were not significant. Therefore, these findings should be interpreted as possible time-related training responses rather than definitive evidence of superior velocity-specific transfer in the ISO group.

Any transfer to 300°·s⁻^1^ testing may reflect improved force production at task-relevant joint angles, enhanced ability to initiate force rapidly from a static position, or improved static-to-dynamic transition capacity. Previous research suggests that isometric training at deeper joint angles may transfer to selected dynamic tasks through changes in neuromuscular coordination or force-production strategy (35, 37), although the extent of velocity transfer remains debated. Because high-velocity contractions, EMG, corticospinal excitability, and motor-unit behavior were not directly assessed, these mechanisms should be interpreted as plausible explanations rather than confirmed adaptations.

These findings suggest that isometric training may support certain dynamic force components despite the absence of movement, particularly in tasks initiated from a static or crouched position. This may be relevant for Kho-Kho players, who frequently need to generate force rapidly from low starting positions. However, given the modest magnitude of the 300°·s⁻^1^ changes and the absence of significant interaction effects, the velocity-transfer findings should remain conservative until confirmed in larger studies.

### Jump and sprint performance

Improvements in vertical jump height were significant within the ISO group and showed a large within-group effect size. The mean change of approximately 4.23 cm may be practically meaningful for field-sport athletes because improved lower-limb explosive performance can support rapid propulsion and jumping actions (38). However, the Time × Group interaction for jump height was not significant, indicating that clear between-group superiority of ISO over DYN was not confirmed. Therefore, the vertical jump result should be interpreted as a promising within-group response rather than definitive evidence of modality-specific superiority.

Both groups improved SLS initial sprint speed, but the Group × Time interaction was not significant. Accordingly, the sprint result should be interpreted as a general pre-post improvement rather than evidence that ISO produced a greater acceleration response than DYN. Average sprint speed also did not improve significantly. These findings support the inclusion of targeted speed-development training alongside strength training when broader sprint-performance enhancement is the goal.

Specific energy and average contact time did not show significant changes. Although small descriptive changes were observed, these outcomes should be interpreted cautiously because of variability and the small pilot sample.

### Neural vs hypertrophic adaptations

Because the intervention lasted only five weeks, large hypertrophic changes are unlikely, although structural adaptations cannot be excluded without direct measurement. The observed improvements may therefore be partly explained by early-phase neural or coordination-related adaptations, such as improved force initiation, motor-unit recruitment efficiency, or static-to-dynamic transition ability (31, 40–42). These mechanisms are consistent with previous isometric training literature and may have contributed to the improvements observed in torque and initial acceleration. However, because the present study did not include direct measures such as EMG, muscle imaging, tendon-property assessment, or corticospinal excitability, these explanations should be interpreted as plausible mechanisms rather than confirmed findings.

### Kho-Kho performance implications

For Kho-Kho athletes, rapid acceleration from static positions, efficient directional changes, and explosive low-stance movements are critical. The present study provides preliminary evidence that RPE-regulated resistance training may support selected performance attributes relevant to these demands. The modality-specific finding was limited to normalized 75° right knee-extension peak torque, whereas initial squat-start speed improved across both groups without a significant Group × Time interaction. Accordingly, initial acceleration should not be attributed specifically to the isometric intervention. These findings remain exploratory and should not be interpreted as evidence that isometric training is broadly superior to dynamic training.

At the same time, dynamic training also demonstrated relevant benefits in selected outcomes, suggesting that both modalities may have practical value depending on the performance quality targeted. In applied Kho-Kho training, isometric and dynamic resistance exercises may therefore be viewed as complementary methods within a periodized program rather than competing approaches. Future training designs may benefit from combining isometric work for static-to-dynamic force production with dynamic and change-of-direction exercises to address the broader movement demands of competitive play.

### Limitations

Several limitations should be considered when interpreting the present findings. First, the absence of a passive non-training control group limits causal inference, as repeated testing, familiarization, normal team training, and natural short-term adaptation may have contributed to the observed improvements. In addition, participants and assessors were not blinded to group allocation, which may have introduced expectation or measurement bias.

Second, the final sample was small, and attrition was considerable. Of the 28 athletes initially recruited, 17 completed the intervention and post-testing; 11 were excluded because of scheduling conflicts or insufficient training attendance. This may have reduced statistical power, increased uncertainty around effect-size estimates, and introduced selection bias. The final sample also included both male and female athletes, but sex-specific effects could not be examined because of the small subgroup sizes.

Third, multiple outcomes were analyzed without formal adjustment for multiple comparisons; therefore, the possibility of false-positive findings cannot be excluded. Although the squat linear sprint and BFS vertical jump reflect relevant Kho-Kho performance components, they do not capture all multidirectional, reactive, and tactical demands of competitive play. Direct neuromuscular mechanisms were also not assessed using EMG, muscle imaging, or tendon-property measurements; therefore, explanations related to neural or structural adaptations remain speculative.

Future studies should include larger samples, passive or active control groups, preferably crossover designs, and assessor blinding where feasible. Longer interventions, direct mechanistic assessments, sex-specific analyses, and additional Kho-Kho-specific tests such as change-of-direction, reactive agility, and chase–dodge tasks would strengthen ecological validity and practical application.

## Conclusion

This pilot study suggests that both isometric and dynamic resistance training, when regulated using perceived exertion, may improve selected neuromuscular and sport–related outcomes in collegiate Kho–Kho athletes over five weeks. A significant modality–specific difference was observed for normalized 75° right knee–extension peak torque, with a larger change in ISO. Initial squat–start speed improved in both groups, but the between–group difference in change was not significant. Vertical jump improvements also did not demonstrate a significant Group × Time interaction. Therefore, definitive superiority of isometric training over dynamic resistance training cannot be concluded from this exploratory pilot study.

From an applied perspective, RPE-regulated isometric training may be a useful short-term option for developing force production from low or semi-static positions, while dynamic resistance training may remain valuable as a complementary method within a periodized Kho-Kho training program. Future studies with larger samples, control conditions, longer interventions, direct neuromuscular measurements, and additional Kho-Kho-specific performance tests are needed to confirm these preliminary findings.

## Data Availability

The raw data supporting the conclusions of this article will be made available by the authors, without undue reservation.
