# Correction: Neuromuscular adaptations to load-matched isometric vs. dynamic resistance training in Kho-Kho athletes: a pilot investigation

**DOI:** 10.3389/fspor.2026.1945223

**Published:** 2026-08-24

**Authors:** Zhaoxiang Zhang, Małgorzata Tomicka, Xiaodan Guo, Mingmao Li, Piotr Aschenbrenner

**Affiliations:** 1 Gdansk University of Physical Education and Sport, Gdańsk, Poland; 2 Department of Biomechanics and Sport Engineering, Gdansk University of Physical Education and Sport, Gdansk, Poland

**Keywords:** Kho-Kho, isometric training, dynamic resistance training, perceived exertion, peak torque, sprint acceleration, vertical jump, reactive strength index

The department name was incorrect in affiliation 2. The correct affiliation appears above.

In the abstract, the article incorrectly reported a significant Group×Time interaction for initial squat-start sprint speed and attributed a greater initial-acceleration response to ISO. The abstract has been corrected to read:

**Introduction:** This pilot study compared the effects of isometric resistance training (ISO) and dynamic resistance training (DYN) on neuromuscular and sport-related performance in collegiate Kho-Kho athletes.

**Methods:** Seventeen athletes (11 males and 6 females) completed the study and were included in the final analysis: ISO, *n* = 9 (6 males and 3 females); DYN, *n* = 8 (5 males and 3 females). Both groups completed a five-week training program, with intensity regulated using modality-specific rating-of-perceived-exertion scales and a target perceived-effort range of 6–7. Pre- and post-intervention assessments included knee extension and flexion torque at multiple angles and velocities, squat linear sprint performance, vertical jump metrics, and reactive strength index.

**Results:** A significant Group×Time interaction was observed for normalized 75° right knee-extension peak torque, F(1,15) = 6.874, p = .019, *η*^2^p = .314. Initial squat-start speed improved across groups, F(1,15) = 20.435, p < .001, *η*^2^p = .577, but the Group×Time interaction was not significant, F(1,15) = 1.083, p = .315, *η*^2^p = .067. Vertical jump improvement did not show a significant Group×Time interaction, and isokinetic torque outcomes showed no clear modality-specific superiority.

**Discussion:** These exploratory findings support a possible short-term, joint-angle-specific strength benefit of RPE-regulated isometric training. However, a modality-specific advantage for sprint acceleration was not supported, and definitive superiority over dynamic resistance training cannot be concluded.

There were mistakes in Table 3 as published. Several descriptive and inferential statistics for normalized isometric peak torque were incorrectly transcribed during table preparation. The corrected Table 3 appears below.

**Table 3:** Normalized isometric peak torque and repeated-measures ANOVA.

Outcome	Group	Change/Z	SD	Within-group p	Effect size	95% CI	Time effect	Group effect	Group×Time interaction
PT 75° L EX	ISO	−0.335	0.189	.001	dz = −1.769	[−2.823, −0.678]	F(1,15) = 17.132 p = .001 *η*2p = .533	F(1,15) = 0.345 p = .566 *η*2p = .022	F(1,15) = 3.378 p = .086 *η*2p = .184
	DYN	−0.129	0.270	.219	d_z_ = −0.477	[−1.197, 0.273]			
PT 75° R EX	ISO	−0.283	0.315	.027	d_z_ = −0.900	[−1.665, −0.097]	F(1,15) = 0.794 p = .387 η^2^p = .050	F(1,15) = 3.280 p = .090 η^2^p = .179	F(1,15) = 6.874 p = .019 η^2^p = .314
	DYN	0.140	0.350	.297	d_z_ = 0.398	[−0.337, 1.108]			
PT 75° L Flex	ISO	−0.252	0.209	.007	d_z_ = −1.207	[−2.061, −0.313]	F(1,15) = 29.528 p < .001 η^2^p = .663	F(1,15) = 0.222 p = .644 η^2^p = .015	F(1,15) = 1.258 p = .280 η^2^p = .077
	DYN	−0.166	0.062	<.001	d_z_ = −2.677	[−4.197, −1.127]			
PT 75° R Flex	ISO	−0.203	0.137	.002	d_z_ = −1.480	[−2.426, −0.494]	F(1,15) = 27.936 p < .001 η^2^p = .651	F(1,15) = 1.086 p = .314 η^2^p = .068	F(1,15) = 0.289 p = .599 η^2^p = .019
	DYN	−0.166	0.151	.017	d_z_ = −1.101	[−1.972, −0.184]			
PT 45° L EX	ISO	0.126	0.286	.221	d_z_ = 0.442	[−0.257, 1.117]	F(1,15) = 1.108 p = .309 η^2^p = .069	F(1,15) = 2.471 p = .137 η^2^p = .141	F(1,15) = 0.317 p = .582 η^2^p = .021
	DYN	Z = −1.400	—	.161	r = .495	—			
PT 45° R EX	ISO	0.041	0.205	.567	d_z_ = 0.199	[−0.467, 0.853]	F(1,15) = 1.906 p = .188 η^2^p = .113	F(1,15) = 0.335 p = .571 η^2^p = .022	F(1,15) = 0.444 p = .515 η^2^p = .029
	DYN	Z = −1.400	—	.161	r = .495	—			
PT 45° L Flex	ISO	−0.343	0.224	.002	d_z_ = −1.536	[−2.503, −0.530]	F(1,15) = 25.002 p < .001 η^2^p = .625	F(1,15) = 0.100 p = .757 η^2^p = .007	F(1,15) = 1.491 p = .241 η^2^p = .090
	DYN	−0.209	0.231	.038	d_z_ = −0.902	[−1.714, −0.047]			
PT 45° R Flex	ISO	−0.291	0.114	<.001	d_z_ = −2.563	[−3.942, −1.155]	F(1,15) = 60.379 p<.001 η^2^p = .801	F(1,15) = 0.082 p = .779 η^2^p = .005	F(1,15) = 3.718 p = .073 η^2^p = .199
	DYN	−0.175	0.134	.008	d_z_ = −1.308	[−2.251, −0.321]			

ISO = isometric resistance training group; DYN = dynamic resistance training group; PT = peak torque; EX = extension; Flex = flexion; ES = effect size; CI = confidence interval; η^2^p = partial eta squared. Change values were calculated as pre-intervention minus post-intervention values; therefore, negative values indicate an increase from pre to post for torque outcomes. For normally distributed outcomes, within-group changes are reported as mean change ± SD with Cohen's d_z and 95% CI. For non-normally distributed outcomes, Wilcoxon Z and effect size r are reported without an unbounded CI.

There were mistakes in Table 4 as published. Several within-group statistics, including Wilcoxon Z, effect size r, change values, and Cohen's d_z, were inadvertently copied or assigned to incorrect rows. The corrected Table 4 appears below.

**Table 4:** Normalized isokinetic peak torque and repeated-measures ANOVA.

Outcome	Group	Change/Z	SD	Within-group p	Effect size	95% CI	Time effect	Group effect	Group×Time interaction
PT 60°/s L EX	ISO	0.194	0.165	.008	dz = 1.172	[0.288, 2.015]	F(1,15) = 14.730 p = .002 η2p = .495	F(1,15) = 0.108 p = .747 η2p = .007	F(1,15) = 0.028 p = .870 η2p = .002
	DYN	0.177	0.232	.067	d_z_ = 0.766	[−0.051, 1.543]			
PT 60°/s R EX	ISO	−0.044	0.195	.519	d_z_ = −0.225	[−0.880, 0.444]	F(1,15) = 0.010 p = .920 η^2^p = .001	F(1,15) = 0.024 p = .879 η^2^p = .002	F(1,15) = 0.373 p = .551 η^2^p = .024
	DYN	0.031	0.308	.781	d_z_ = 0.102	[−0.596, 0.794]			
PT 60°/s L Flex	ISO	0.033	0.146	.519	d_z_ = 0.225	[−0.444, 0.880]	F(1,15) = 0.485 p = .497 η^2^p = .031	F(1,15) = 0.205 p = .657 η^2^p = .013	F(1,15) = 2.586 p = .129 η^2^p = .147
	DYN	−0.083	0.150	.163	d_z_ = −0.551	[−1.283, 0.214]			
PT 60°/s R Flex	ISO	−0.047	0.091	.162	d_z_ = −0.514	[−1.199, 0.199]	F(1,15) = 3.842 p = .069 η^2^p = .204	F(1,15) = 0.020 p = .888 η^2^p = .001	F(1,15) = 0.104 p = .752 η^2^p = .007
	DYN	Z = −1.120	—	.263	r = .396	—			
PT 300°/s L EX	ISO	−0.015	0.102	.665	d_z_ = −0.150	[−0.803, 0.512]	F(1,15) = 1.934 p = .185 η^2^p = .114	F(1,15) = 0.824 p = .378 η^2^p = .052	F(1,15) = 0.584 p = .457 η^2^p = .037
	DYN	−0.053	0.099	.175	d_z_ = −0.533	[−1.262, 0.228]			
PT 300°/s R EX	ISO	0.001	0.089	.977	d_z_ = 0.010	[−0.644, 0.663]	F(1,15) = 1.982 p = .180 η^2^p = .117	F(1,15) = 0.169 p = .686 η^2^p = .011	F(1,15) = 2.089 p = .169 η^2^p = .122
	DYN	−0.066	0.102	.110	d_z_ = −0.646	[−1.397, 0.140]			
PT 300°/s L Flex	ISO	−0.063	0.078	.042	d_z_ = −0.805	[−1.547, −0.027]	F(1,15) = 8.219 p = .012 η^2^p = .354	F(1,15) = 1.630 p = .221 η^2^p = .098	F(1,15) = 0.594 p = .453 η^2^p = .038
	DYN	−0.036	0.062	.144	d_z_ = −0.582	[−1.320, 0.190]			
PT 300°/s R Flex	ISO	−0.096	0.105	.025	d_z_ = −0.916	[−1.686, −0.109]	F(1,15) = 11.904 p = .004 η^2^p = .442	F(1,15) = 1.098 p = .311 η^2^p = .068	F(1,15) = 0.460 p = .508 η^2^p = .030
	DYN	−0.065	0.084	.067	d_z_ = −0.766	[−1.542, 0.051]			

ISO = isometric resistance training group; DYN = dynamic resistance training group; PT = peak torque; EX = extension; Flex = flexion; ES = effect size; CI = confidence interval; η^2^p = partial eta squared. Change values were calculated as pre-intervention minus post-intervention values. Cohen's d_z and 95% CI are reported for parametric comparisons; Wilcoxon Z and r are reported for nonparametric comparisons.

There were mistakes in Table 6 as published. Statistical values for the Time, Group, and Group×Time effects were placed in incorrect effect columns, and several within-group statistics were incorrectly assigned during manuscript preparation. The corrected Table 6 appears below.

**Table 6:** Squat linear sprint outcomes and repeated-measures ANOVA summary.

Outcome	Group	Change/Z	SD	Within-group p	Effect size	95% CI	Time effect	Group effect	Group×Time interaction
Initial speed (m/s)	ISO	−0.721	0.567	.005	dz = −1.271	[−2.146, −0.356]	F(1,15) = 20.435; p < .001; η2p = .577	F(1,15) = 0.961; p = .342; η2p = .060	F(1,15) = 1.083; p = .315; η2p = .067
	DYN	−0.451	0.493	.036	d_z_ = −0.916	[−1.732, −0.057]			
Average speed (m/s)	ISO	−0.177	0.329	.145	d_z_ = −0.538	[−1.226, 0.179]	F(1,15) = 1.170; p = .296; η^2^p = .072	F(1,15) = 0.051; p = .824; η^2^p = .003	F(1,15) = 2.129; p = .165; η^2^p = .124
	DYN	0.026	0.228	.754	d_z_ = 0.115	[−0.584, 0.806]			
Specific energy (J/kg)	ISO	Z = −0.652	—	.515	r = .217	—	F(1,15) = 0.001; p = .971; η^2^p < .001	F(1,15) = 0.768; p = .395; η^2^p = .049	F(1,15) = 0.493; p = .493; η^2^p = .032
	DYN	0.034	0.129	.475	d_z_ = 0.267	[−0.449, 0.964]			
Average contact time (s)	ISO	Z = −0.830	—	.407	r = .277	—	F(1,15) = 0.698; p = .417; η^2^p = .044	F(1,15) = 0.076; p = .787; η^2^p = .005	F(1,15) = 1.321; p = .268; η^2^p = .081
	DYN	Z = −1.400	—	.161	r = .495	—			

ISO = isometric resistance training group; DYN = dynamic resistance training group; ES = effect size; CI = confidence interval; η^2^p = partial eta squared. Change values were calculated as pre-intervention minus post-intervention values. Negative initial-speed changes therefore indicate higher post-intervention speed.

There was a mistake in Figure 4 as published. The figure and caption did not accurately represent the corrected standardized within-group effects. The corrected Figure 4, based on the reanalyzed torque and sprint data and the available published jump-summary statistics, appears below.

**Figure 4:**
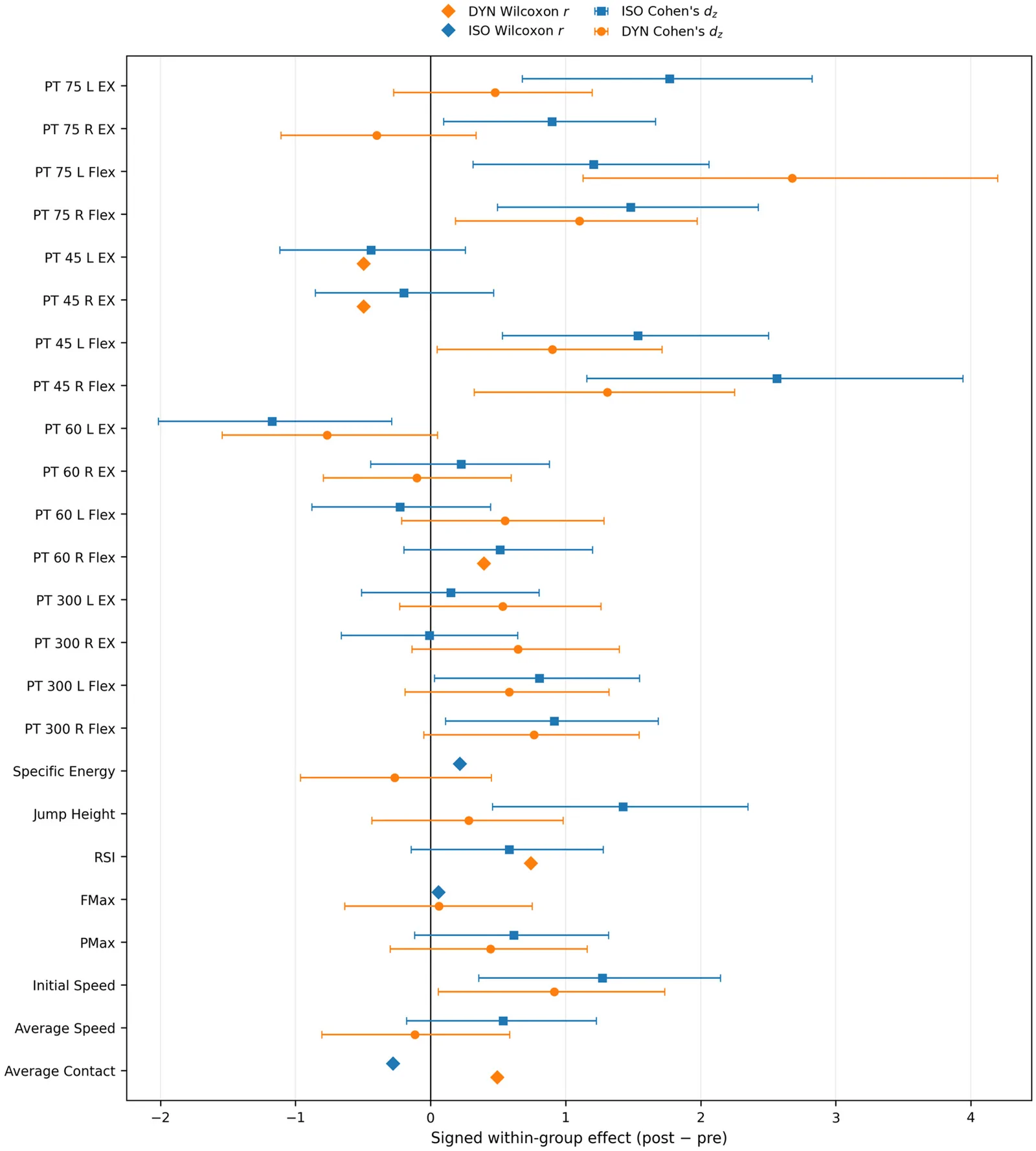
Signed within-group effects for the Isometric (ISO) and Dynamic (DYN) groups. For outcomes analyzed parametrically, Cohen's d_z was calculated as (post-intervention − pre-intervention)/SD of the paired change and is shown with 95% confidence intervals. Outcomes analyzed with Wilcoxon signed-rank tests are shown as signed effect size r without confidence intervals. The sign indicates the direction of the post-minus-pre change; the magnitude of r is not directly comparable with d_z. The jump-height, RSI, FMax, and PMax effect estimates were retained from the published summary statistics because participant-level jump data were unavailable for re-estimation. The plot is descriptive; modality-specific differences should be judged from the Group × Time interaction tests.

A correction has been made to the section **Results**, “Isometric peak torque”, paragraph 1. The published text reported incorrect descriptive and ANOVA values for selected isometric peak-torque outcomes. The correct paragraph reads:

“For isometric testing, normalized peak torque (NPT) results are summarized in Table 3 and illustrated in Figure 2. At 75° knee extension, the ISO group improved on the left (−0.335 ± 0.189, p = .001, d_z = −1.769) and right (−0.283 ± 0.315, p = .027, d_z = −0.900), whereas the DYN group did not show significant within-group changes. For PT 75° L EX, the main effect of Time was significant, F(1,15) = 17.132, p = .001, η^2^p = .533, whereas the Group×Time interaction was not significant, F(1,15) = 3.378, p = .086, η^2^p = .184. For PT 75° R EX, the Group×Time interaction was significant, F(1,15) = 6.874, p = .019, η^2^p = .314, indicating a larger change in ISO than DYN. At 45° knee extension, no significant Time, Group, or Group×Time effects were observed.”

A correction has been made to the section **Results**, “Peak torque between groups”. This section contained the superseded interaction statistics from Table 3. The corrected section reads:

“Between-group effects for isometric NPT outcomes are presented in Table 3. The only statistically significant Time×Group interaction was observed for PT 75° R EX, F(1,15) = 6.874, p = .019, η^2^p = .314, indicating that the magnitude of change differed between ISO and DYN for this outcome. PT 75° L EX showed a non-significant trend toward a Time×Group interaction, F(1,15) = 3.378, p = .086, η^2^p = .184. Similarly, PT 45° R Flex showed a large but non-significant interaction effect, F(1,15) = 3.718, p = .073, η^2^p = .199. Therefore, although several outcomes showed significant within-group or Time effects, clear modality-specific superiority was supported only for PT 75° R EX.”

A correction has been made to the section **Results**, “Squat linear sprint”. The text incorrectly reported a significant Group×Time interaction for initial squat-start speed and duplicated several ANOVA values across effect columns. The corrected section reads:

“Sprint performance results are presented in Table 6. Both groups demonstrated significant within-group improvements in initial speed from the squat-start position (ISO: −0.721 ± 0.567 m/s, p = .005, d_z = −1.271; DYN: −0.451 ± 0.493 m/s, p = .036, d_z = −0.916). The main effect of Time was significant, F(1,15) = 20.435, p < .001, η^2^p = .577, indicating an overall pre–post improvement. However, neither the main effect of Group, F(1,15) = 0.961, p = .342, η^2^p = .060, nor the Group×Time interaction, F(1,15) = 1.083, p = .315, η^2^p = .067, was significant. Therefore, the data support improvement in both groups but do not support a modality-specific advantage for initial squat-start speed.

“No significant Time, Group, or Group×Time effects were observed for average sprint speed, specific energy, or average contact time. For average speed, the Time effect was F(1,15) = 1.170, p = .296, η^2^p = .072, and the Group×Time interaction was F(1,15) = 2.129, p = .165, η^2^p = .124. For specific energy, the Group×Time interaction was F(1,15) = 0.493, p = .493, η^2^p = .032; for average contact time, it was F(1,15) = 1.321, p = .268, η^2^p = .081. Thus, the sprint findings indicate a general improvement in initial speed across groups, whereas average speed, specific energy, and contact time were not significantly altered.”

A correction has been made to the section **Results**, “Standardized mean difference (SMD)”. The interpretation of Figure 4 incorporated the incorrect sprint interaction and treated the plotted effects as a single metric. The corrected section reads

“Figure 4 presents signed within-group effects for the ISO and DYN groups. For parametrically analyzed outcomes, points represent Cohen's d_z calculated as (post-intervention−pre-intervention)/SD of the paired change, with 95% confidence intervals. Outcomes analyzed with Wilcoxon signed-rank tests are shown as signed r values without confidence intervals. Positive values indicate an increase from pre to post, whereas negative values indicate a decrease; the practical meaning of the direction depends on the outcome. The jump-height, RSI, FMax, and PMax points are retained from the published summary statistics because the participant-level jump dataset was not available for re-estimation. Because r and d_z are different effect-size metrics, their magnitudes should not be compared directly.

“The plot is descriptive and must be interpreted together with the Group×Time interaction tests. The corrected analyses support a differential response for PT 75° R EX. Initial squat-start speed improved in both groups without a significant Group×Time interaction, and vertical jump improvement likewise did not show a significant Group×Time interaction. Therefore, Figure 4 does not establish modality-specific superiority for sprint or jump performance.”

A correction has been made to the section **Discussion**, “Jump and sprint performance”, paragraph 2. The discussion incorrectly interpreted the sprint result as evidence of a differential training response. The corrected paragraph reads:

“Both groups improved SLS initial sprint speed, but the Group×Time interaction was not significant. Accordingly, the sprint result should be interpreted as a general pre–post improvement rather than evidence that ISO produced a greater acceleration response than DYN. Average sprint speed also did not improve significantly. These findings support the inclusion of targeted speed-development training alongside strength training when broader sprint-performance enhancement is the goal.”

A correction has been made to the section **Discussion**, “Kho-Kho performance implications”, paragraph 1. The published discussion attributed the initial-acceleration result specifically to isometric training. The corrected paragraph reads:

“For Kho-Kho athletes, rapid acceleration from static positions, efficient directional changes, and explosive low-stance movements are critical. The present study provides preliminary evidence that RPE-regulated resistance training may support selected performance attributes relevant to these demands. The modality-specific finding was limited to normalized 75° right knee-extension peak torque, whereas initial squat-start speed improved across both groups without a significant Group×Time interaction. Accordingly, initial acceleration should not be attributed specifically to the isometric intervention. These findings remain exploratory and should not be interpreted as evidence that isometric training is broadly superior to dynamic training.”

A correction has been made to the section **Conclusion**, paragraph 1. The conclusion was revised to remove the unsupported claim of a modality-specific advantage for initial sprint acceleration. The corrected paragraph reads:

“This pilot study suggests that both isometric and dynamic resistance training, when regulated using perceived exertion, may improve selected neuromuscular and sport-related outcomes in collegiate Kho-Kho athletes over five weeks. A significant modality-specific difference was observed for normalized 75° right knee-extension peak torque, with a larger change in ISO. Initial squat-start speed improved in both groups, but the between-group difference in change was not significant. Vertical jump improvements also did not demonstrate a significant Group×Time interaction. Therefore, definitive superiority of isometric training over dynamic resistance training cannot be concluded from this exploratory pilot study.”

The original version of this article has been updated.

